# Characterization of the Pathology, Biochemistry, and Immune Response in Kunming (KM) Mice Following *Fasciola gigantica* Infection

**DOI:** 10.3389/fcimb.2021.793571

**Published:** 2022-01-05

**Authors:** Xuefang Mei, Yaoyao Zhang, Chenyu Quan, Yiying Liang, Weiyi Huang, Wei Shi

**Affiliations:** ^1^ Xinxiang Key Laboratory of Pathogenic Biology, Department of Pathogenic Biology, School of Basic Medical Sciences, Xinxiang Medical University, Xinxiang, China; ^2^ School of Animal Science and Technology, Guangxi University, Nanning, China; ^3^ Guangxi Key Laboratory of Veterinary Biotechnology, Guangxi Veterinary Research Institute, Nanning, China; ^4^ School of Preclinical Medicine, Guangxi Medical University, Nanning, China

**Keywords:** *Fasciola gigantica*, mice, pathology, immunology, biochemical indices

## Abstract

As a putative model of *Fasciola gigantica* infection, detailed data in Kunming (KM) mice infected with *F. gigantica* are lacking. In this study, KM mice were orally infected with 15 metacercaria for 8 weeks. Macroscopic and microscopic changes, serum biochemistry, cytokine responses, and changes in parasite-specific immunoglobulin G (IgG) antibody levels were monitored at 1, 3, 5, 7, and 8 weeks post-infection (wpi), respectively. The serum levels of aspartate aminotransferase (AST) and alanine aminotransferase (ALT) increased after infection, while that of albumin (ALB) decreased, which was positively correlated with the degree of liver damage. Between 5 and 7 wpi, the mice showed symptoms of anemia and weight loss, possibly caused by the decrease of alkaline phosphatase (ALP). Moreover, the changing tendencies of the levels of globulin (GLB) and parasite-specific IgG antibody were similar, suggesting a potential correlation between GLB production and adaptive immune response in the host. Coordinated variations in interferon gamma (IFN-γ) and interleukin 4 (IL-4) indicated a mixed T helper 1 (Th1)/Th2 cellular immune response. Furthermore, the serum IgG antibody increased after infection and peaked at 5 wpi, and it was positively correlated with the average parasite burdens. The worms collected from mice were approximately 1 cm in length at 8 wpi, their digestive and reproductive systems were well developed, and no eggs were found in the uterus. To the best of our knowledge, this is the first report describing detailed histological, biochemical, and immunological indices in KM mice infected with *F. gigantica*, which provides basic information on KM mice against infection with *F. gigantica.*

## Introduction

Fasciolosis is a globally distributed foodborne, zoonotic parasitic disease caused by *Fasciola hepatica* or *Fasciola gigantica* (Ahasan et al., 2016; Cabada et al., 2016). The life cycle of *Fasciola* includes the following stages: egg, miracidium, sporocyst, redia, cercaria, metacercaria, excystic juvenile, and adult parasite (Saba and Korkmaz, 2005; Mcmanus and Dalton, 2007). The egg, miracidium, sporocyst, redia, cercaria, and metacercaria stages occur in microorganisms that are usually detected with a microscope. The larval stage requires a molluscan intermediate host such as freshwater snails, while in the adult stage, the parasite lives on the terminal mammalian host (Roy and Reddy, 1969; Halton and Johnston, 1983). Ruminants, including sheep and cattle, are the natural hosts of *Fasciola*. Yet, these animal models are not suitable for research purposes due to the high cost of maintenance (experimental site, food, and shelter) and the complexity of the experimental protocols, which can seriously hinder experimentation in this field.

Small laboratory animals such as rabbits, mice, and rats are common animal models used to study *F. hepatica* infection. However, different animal models show varied susceptibilities to *Fasciola* (Dixon, 1964; Sewell, 1966; Reddington et al., 1986). Different parasite burdens, even within the same host species, can cause diverse pathological changes, immune responses, and parasite recovery rates (Kendall and Parfitt, 1962; Boray, 1967).

Murine models, such as mice and rats, are easy to handle and are not too costly to maintain. Therefore, using a murine laboratory model for experiments of *F. gigantica* infection may largely overcome the limitations of using large animals. BALB/c mice, Kunming (KM) mice, C57BL/6 mice, and Swiss mice are widely used models for biological and biomedical research. However, comparative studies have confirmed that the conditions of pathogen infection could differ when using different mouse strains (Jussi et al., 2005; Ley et al., 2005). In China, KM mice are the most productive and the most preferably used mouse strain for laboratory research purposes, including vaccine or drug studies (Liu et al., 2004; Zhang et al., 2007; Yuan et al., 2011). These mice are the outbred offspring of Swiss mice that have been bred into different inbred lines in different regions. Their advantages compared to Swiss mice include disease resistance, adaptability, high reproductive rate, and high survival rate (Shang et al., 2009). These advantages make them ideal animal models for artificial infection with *F. gigantica*.

Previous studies have shown that small laboratory animals may be potentially used for early infection studies (particularly the migrating larvae stage) of larger helminthic parasites (Mango et al., 1972; Dawkins and Grove, 1982; Kozek and Marroquin, 1982; Eriksen et al., 1987). The migrating larvae stage is considered as the best time to eliminate liver flukes (Kaplan and Ray, 2001). However, data on the immunology, biochemistry, and pathology of early infection of *F. gigantica* in KM mice are still lacking. In addition, there are no existing data that can serve as guidelines as to what can be expected during infection, which are all critical for further investigation when using this animal as a model and for the comparison of this model to other animal models, as well as for any downstream research on vaccine and therapeutic reagent development.

In this study, we investigated the dynamic changes in the pathology, serum biochemistry, and T helper 1 (Th1)/Th2 immune responses in KM mice infected with *F. gigantica* metacercaria during the early infection stage. The connection between macroscopic and microscopic changes was also investigated. These data may further explain the relationship between the parasite and the host. 

## Materials and Methods

### Preparation of *F. gigantica* Metacercaria

*F. gigantica* metacercaria was prepared as previously described (Anuracpreeda et al., 2009). Briefly, the parasite eggs were obtained from the gall bladder of infected water buffalo from a local slaughterhouse using the nylon mesh elutriation method. The eggs were washed and then incubated in a 28°C incubator for approximately 12–14 days. The hatched miracidia were isolated and used to infect naive freshwater snails (*Galba pervia*) locally collected from ditches and water tunnels bordering paddy fields, with a parasite load of two miracidia per snail in the tanks. Plastic films were cut into small pieces and placed over the water surface to collect the metacercaria. After approximately 35 days, the metacercaria attached to the films were collected, rinsed, counted, and stored in sterilized water at 4°C until further use.

### Animals, Experimental Infection, and Sampling

Six-week-old female KM mice (SPF grade, 20–25 g body weight) were provided by the Experimental Animal Center of Guangxi Medical University. In order to explore the appropriate number of metacercaria infections, the infection cycle, and the development of *F. gigantica* during infection, 144 KM mice were divided into four groups. Each group was infected with 5, 15, 30, and 50 metacercariae by oral gavage. From 1 to 9 weeks post-infection (wpi), the clinical symptoms and the number of deaths were recorded, and fecal eggs were monitored. Four mice from each group were sacrificed and dissected every week, after which worms were collected and analyzed. Another four mice served as uninfected controls for observation of the clinical symptoms from 1 to 9 wpi.

Sixty KM mice were randomly assigned into the infection group (40 animals) and the control group (20 animals). Mice in the infected group received 15 metacercaria (the dose of infection was based on the screening results) in 0.5 ml sterilized water by oral gavage, while mice in the control group were orally administered an equal volume of water. Four mice from each group were euthanized at 1, 3, 5, 7, and 8 wpi. Blood was collected by retro-orbital bleeding for serum separation before the mice were sacrificed. The livers were then harvested and analyzed by histopathology. Commercial feed and sterilized water were provided *ad libitum* for all animals during the study period. All mice were examined weekly to monitor signs of infection.

### Gross Examination, Parasite Burdens, and Macroscopic Liver Lesion

All mice were sacrificed and examined for gross pathological lesions. Successful infection by *F. gigantica* was confirmed by the observation of typical pathological lesions. The organs and tissues (including subcutaneous tissue, brain, heart, lung, stomach, liver, gallbladder, intestine, and urinary bladder) were individually separated, and visible parasites were examined by the naked eye so as not to miss any possible ectopic parasitism of liver flukes. In order to facilitate statistical analysis of the correlation between lesions and the various biochemical and immunological indices, the severity of liver lesions was scored (from 0 to 5 points) according to the criteria of Lvova et al. (2012) and Raadsma et al. (2007), with minor modifications: 0 point, no obvious tissue necrosis or liver nodules; 1 point, mild liver necrosis or the presence of nodules, with lesions occupying <5% of the surface of the liver; 2 points, damaged liver area <15%; 3 points, liver damage or nodule area of <30%; 4 points, severe liver injury or liver nodules, with lesion area <50%; and 5 points, extensive liver necrosis, present in >50% of the liver surface.

### Histopathological Evaluation

The liver tissue was preserved in Bouin’s fixative for 2 days, after which it was dehydrated, rinsed in xylene, and embedded in paraffin. Three-micrometer ultrathin sections of the paraffin-embedded tissue were mounted onto glass slides and stained with hematoxylin and eosin (H&E) dye. The samples were then sealed and the stained tissue sections were microscopically examined at ×400 magnification and imaged using a Zeiss Axio Imager manual upright research microscope (HITACHI, Tokyo, Japan).

### Measurements of Biochemical Indices and Cytokines

To evaluate the liver function, several enzymes in the serum were examined. Sera from all mice were separated from whole coagulated blood by centrifugation. Subsequently, routine biochemical indices, including aspartate aminotransferase (AST), alanine aminotransferase (ALT), alkaline phosphatase (ALP), albumin (ALB), and globulin (GLB), were determined using commercial diagnostic kits (Nanjing Jiancheng Bioengineering Institute, Jiangsu, China) and an automatic biochemical analyzer (HITACHI, Tokyo, Japan). The serum levels of the circulating cytokines interferon gamma (IFN-γ) and interleukin 4 (IL-4) were measured using commercial enzyme-linked immunosorbent assay (ELISA) kits (Cloud-Clone Corp., Houston, TX, USA) following the manufacturer’s instructions.

### Detection of *F. gigantica*-Specific IgG Antibody

ELISA was performed to assess the dynamics of antibody titer against *F. gigantica* infection according to the protocol of Ridi et al. (2007). Briefly, *F. gigantica* excretory/secretory products (FgESPs) were prepared following the previously described procedure (Ridi et al., 2007). Flat-bottom 96-well microtiter plates (Jet Biofil, Guangzhou, China) were coated with 0.25 mg/well of laboratory-made FgESPs in carbonate–bicarbonate buffer, pH 9.6, overnight at 4°C. The tested serum was diluted 1:200 with PBST [0.05 M phosphate-buffered saline containing 0.05% (*v*/*v*) Tween 20], followed by incubation for 1 h at 37°C. All experiments were performed in duplicate. All wells were washed with PBST four times and incubated for another 1 h at 37°C with 1:20,000-diluted goat anti-mouse immunoglobulin G (IgG) (CWBIO, Beijing, China). After washing with PBST four times, bound antibodies were detected by adding 100 μl/well of tetramethylbenzidine (CWBIO, Beijing, China) for 20 min at 37°C. Absorbance was measured at 450 nm on a microplate reader (iMarkTM Microplate Reader, Bio-Rad, Hercules, CA, USA).

### Statistical Analysis

Statistical analysis and graphing were performed using GraphPad Prism version 6.02 (GraphPad Software Inc., La Jolla, CA, USA). The levels of all biochemical indices, cytokines, and IgG antibody titers were compared at different time points after infection using one-way analysis of variance (ANOVA) with *post-hoc* least significant difference (LSD) *t*-tests. Pearson’s correlation coefficient (*r* value) was used to assess the correlation between the average parasite burdens, liver lesion scores, and the serum levels of specific IgG antibodies, biochemical indices, and cytokines in a pairwise fashion followed by a two-tailed *post-hoc* test and presented as a *p*-value. All data shown represent the mean ± SEM. The level of significance for all analyses was evaluated with a confidence interval >95% (*p* < 0.05). 

## Results

### Screening of Metacercariae Number Infected in Mice

**Figure 1:**
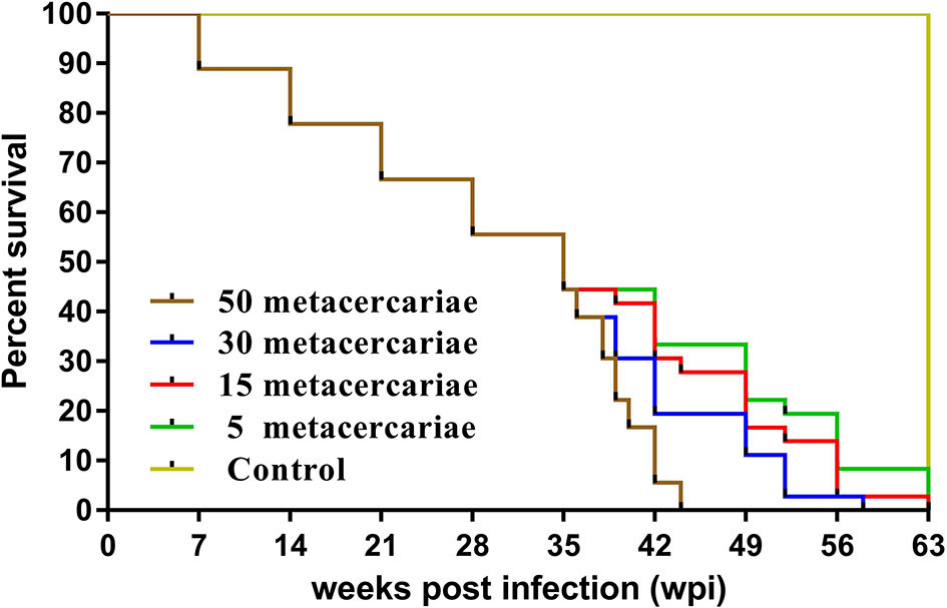
Survival curve of mice. *Green*, *red*, *blue*, and *brown lines* indicate mice infected with 5, 15, 30, and 50 metacercariae, respectively. The *yellow line* represents uninfected mice.

The number of deaths in each group were recorded, including weekly dissection and sudden death (**Figure 1**). Uninfected mice had normal hair, breathing, and feeding during the experiment. Mice infected with 30 and 50 metacercariae showed symptoms of depression, anemia, and abdominal edema at 4 wpi. The longest survival times were 58 and 44 days, respectively. Mice infected with five metacercariae were healthy; only one mouse died after 52 days. No abnormalities were observed in the tissues or organs, and no worm was found at 9 wpi, suggesting that infection of mice with five metacercariae had a low success rate. The disease process of the mice infected with 15 metacercariae was relatively mild, and the worms were collected at all tested time points, except at 1 wpi.

**Figure 2:**
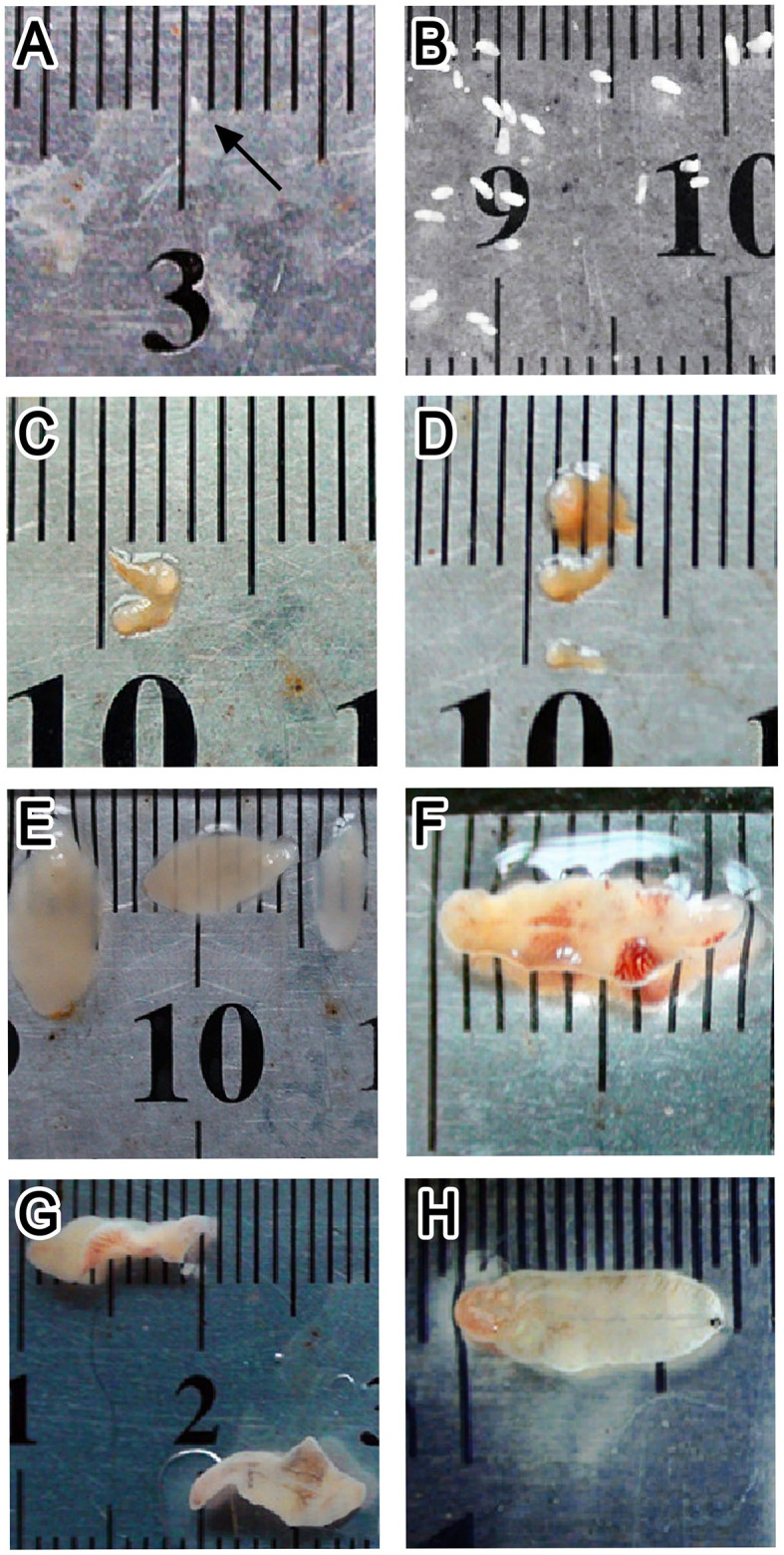
Worms collected from Kunming (KM) mice at different infection stages. **(A–H)** Worms at 1, 2, 3, 4, 5, 6, 7, and 9 weeks post-infection, respectively.

The worms collected from mice were milky white, with a body length of about 0.5 mm at 1 wpi, and intestinal branches were visible under an optical microscope. From 5 weeks after infection, worms were found in the bile ducts of mice; the digestive and reproductive systems of the worms were well developed, and no eggs were found in the uterus. Between 1 and 7 wpi, the length of the worms rapidly increased from <1 mm to about 1 cm. No significant changes in body size and development were observed in the time that followed (**Figure 2**). Therefore, 15 doses of metacercariae were selected as the appropriate dose of infection in KM mice in this study, and the infection period the ranged from 0 to 8 weeks, which was suitable for the study of the juvenile stage of *F. gigantica*.

### Average Parasite Burdens and Macroscopic Liver Lesion Scores

*F. gigantica* was recovered at different time points, except for 1 wpi, and the worm burden reached a peak at 5 wpi, when an average of 2.5 parasites per mice was recovered (**Table 1**). Histopathological evaluation was individually scored (**Table 2**), and the most severe liver lesions were observed at 7 wpi, suggesting that the severity of pathology was time-dependent.

**Table 1:** Average parasite burdens of Kunming (KM) mice infected with *Fasciola gigantica* metacercariae.

Week post-infection (wpi)	1	3	5	7	8
Average number of parasites recovered	0	0.5	2.5	1.75	1.5

**Table 2:** Macroscopic liver lesion scores of Kunming (KM) mice infected with *Fasciola gigantica* metacercariae.

Week post-infection (wpi)	Animal 1	Animal 2	Animal 3	Animal 4
1	0	0	0	0
3	1	0	1	0
5	1	2	1	1
7	4	2	3	2
8	2	2	4	2

A score of 0 indicates no signs of tissue necrosis or liver nodules, a score of 1 indicates mild hepatic necrosis or nodules that are limited to <5% of the liver surface area, 2 indicates liver damage due to damage occupying <15% of the liver surface area, 3 indicates that moderate liver damage is present and nodules are limited to <30% of the liver surface area, 4 represents liver damage and nodules occupying <50% of the liver surface area, and a score of 5 represents extensive liver necrosis and >50% of the liver surface area occupied by liver nodules.

### Gross Lesion and Histopathology

**Figure 3:**
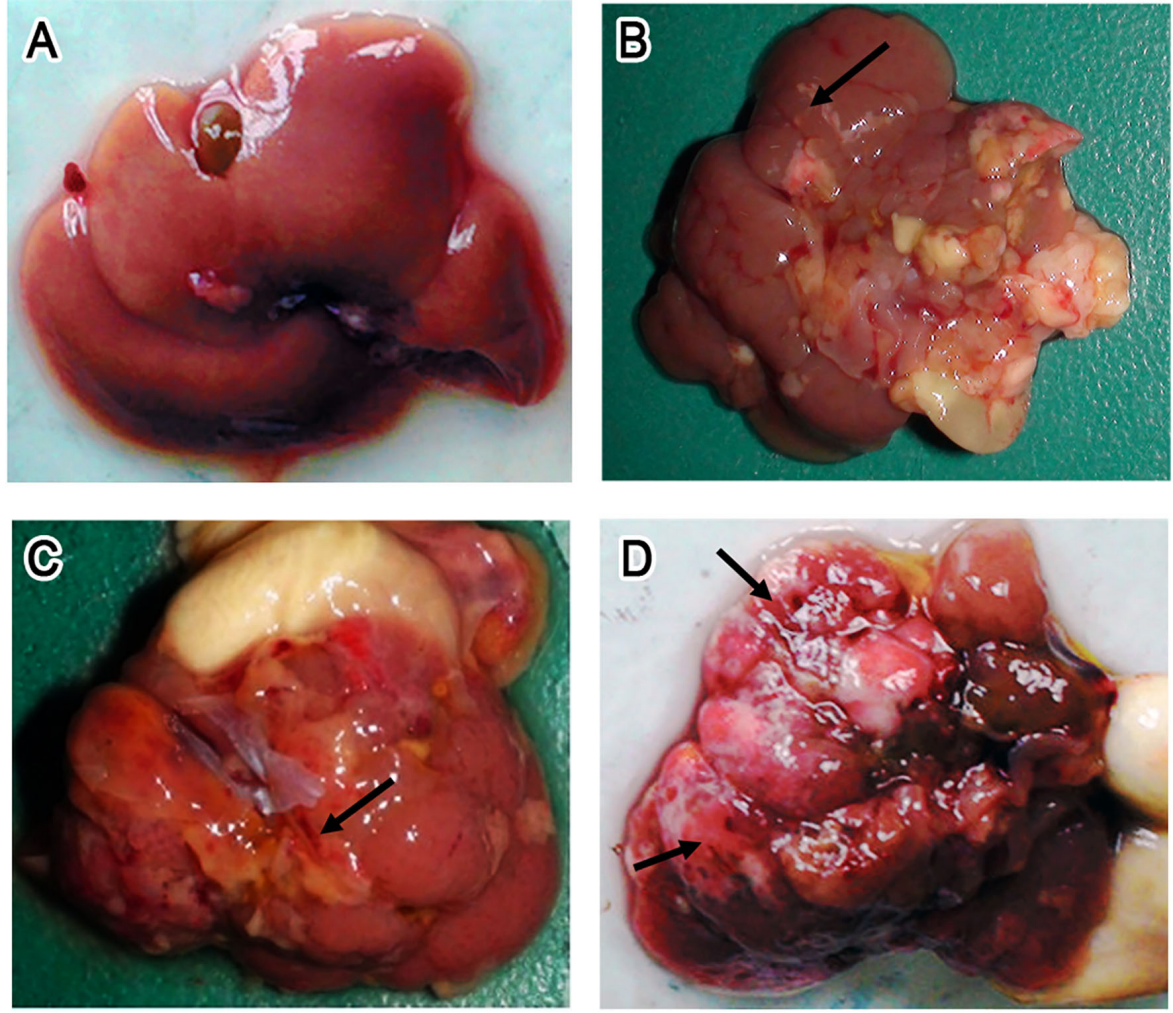
Gross lesion of the liver. **(A–D)** Photographs of livers isolated from uninfected control Kunming (KM) mice **(A)** and mice infected with 15 *Fasciola gigantica* metacercaria at 7 days post-infection (dpi) **(B)** or 49 dpi **(C, D)**. *Black arrows* point to regions with bleeding, while *blue arrows* point to a typical lesion, i.e., gray–white nodules or pyogenic foci in the liver.

Visual examination of control KM mice showed that the liver was dark red and had an overall smooth surface (**Figure 3A**). About 1 week after infection, white spots or streaks of blood became visible on the surface (**Figure 3B**), after which the liver damage gradually worsened. During the late stages of infection (7 wpi), cellulose exudate, connective tissue hyperplasia, and abscesses were observed on the surface (**Figure 3C**). In more severe cases, holes could be observed on the surface and a cellulose-like exudate was visible on the liver serosa. In addition, the texture of the liver appeared tough, and the lobes were no longer clearly defined (**Figure 3D**).

**Figure 4:**
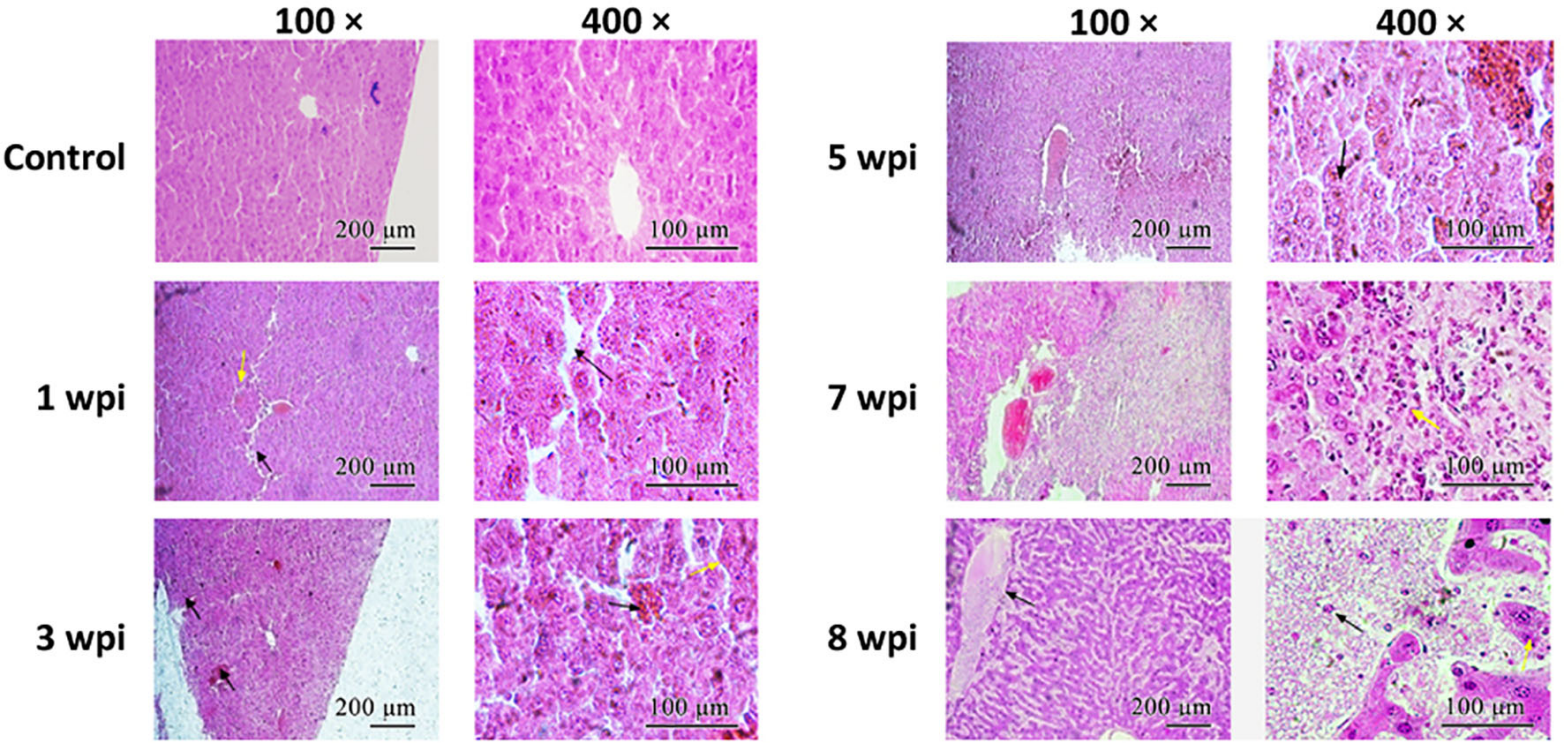
Histopathological characteristics of the liver. The tissue sections were stained with hematoxylin–eosin. *Arrows* indicate the corresponding morphological features described in the article. *Scale bars*: *left panel*, 200 μm; *right panel*, 100 μm.

H&E staining showed that the lobule structure in the control group liver was intact, the hepatocytes were neatly arranged, and the hepatic cord contained radially arranged cells (**Figure 4**). At 1 wpi, the arrangement of hepatocytes became disordered. At 3 wpi, the hepatocytes showed necrosis and disintegration. At 5 wpi, the liver tissue structure was further destroyed, and red blood cells, eosinophils, and lymphocytes significantly increased in the sinusoids. At 7 wpi, multiple focal areas of necrosis could be observed in hepatic structures, while necrosis and structural disintegration, hepatocyte atrophy, and fibroblast proliferation in the portal area were observed in the liver. At 8 wpi, the central vein was filled with red blood cells; some of the red blood cells collapsed, while connective tissues increased in the portal area and a great amount of inflammatory cell infiltration was observed.

### Biochemical Response

Compared with the control group, the serum ALT and AST levels in the experimental group increased at 3 wpi (*p* < 0.01), reaching a peak at 7 wpi. The ALP level significantly increased at 3 wpi (*p* < 0.01) and significantly decreased from 5 to 7 wpi (*p* < 0.01). At 8 wpi, the ALP level increased compared to the levels observed in the control group (*p* > 0.05).

**Figure 5:**
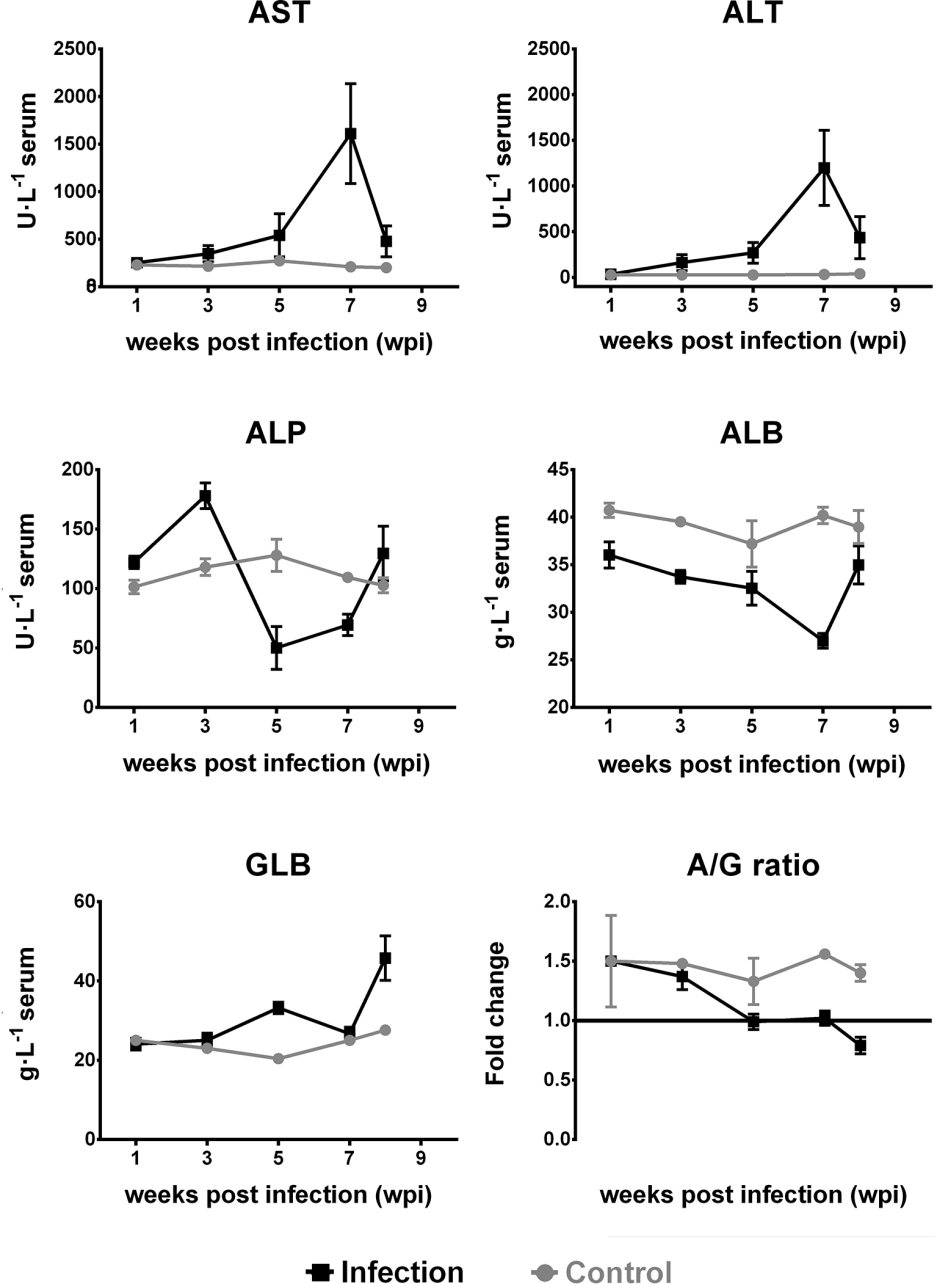
Dynamic changes in serum alanine aminotransferase (ALT), aspartate aminotransferase (AST), alkaline phosphatase (ALP), albumin (ALB), and globulin (GLB) levels and the ratio of ALB to GLB (*A*/*G*) in Kunming (KM) mice infected with *Fasciola gigantica*. The *X*-axis represents weeks post-infection (wpi) and the *Y*-axis represents the biochemical indices or the fold change of the level of ALB level relative to that of GLB. *Red lines* show the trends of the tested biochemical indices in *F. gigantica*-infected mice, while *blue lines* show the trends of the uninfected control mice. The mean values of the biochemical indices from three independent experiments are also plotted.

The level of ALB was lower than that of the control group, showing a downward trend and reaching its lowest value at 7 wpi (*p* < 0.01). The GLB level began to increase at 5 wpi (*p* < 0.01). Between 5 and 8 wpi, the ratio of ALB to GLB (*A*/*G*) was significantly lower when compared to that in the control group (*p* < 0.01; **Figure 5**).

### Th1/Th2 Immune Response

**Figure 6:**
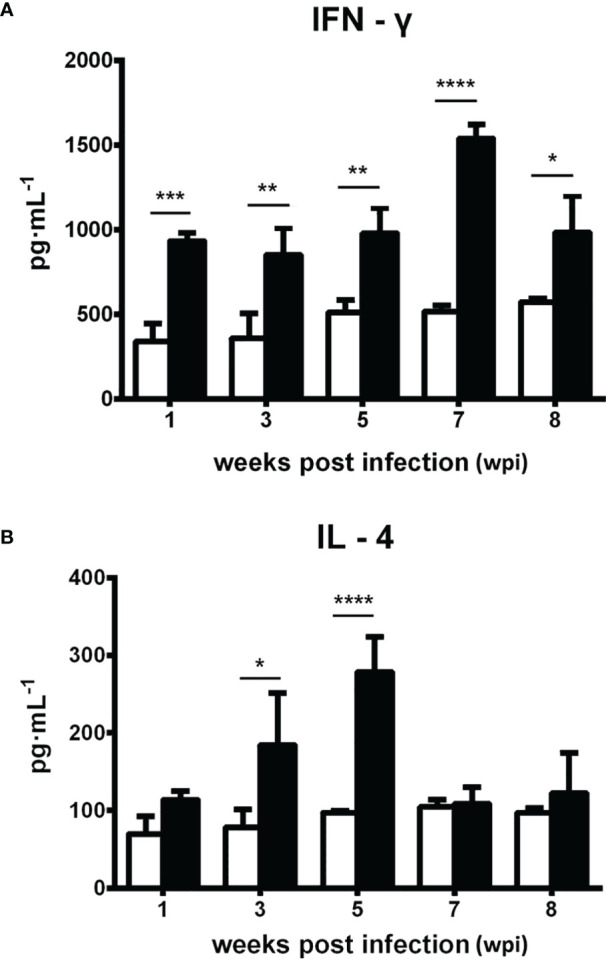
Dynamic changes of the serum cytokines IFN-γ and IL-4 in Kunming (KM) mice infected with *Fasciola gigantica* compared with uninfected control mice. The mean ± SE values of three independent experiments are shown. The *X*-axis represents weeks post-infection (wpi) and the *Y*-axis represents the production of serum cytokine measured by commercial ELISA kits. Significant differences of each time point compared with the control: *p < 0.05; **p < 0.01; ***p < 0.001; ****p < 0.0001 (analyzed by one-way ANOVA, *post-hoc* LSD test).

The level of serum IFN-γ significantly increased at 1 wpi (*p* < 0.001) and remained relatively high, reaching a peak at 7 wpi (*p* < 0.0001), whereas serum IL-4 concentration was elevated from 3 to 5 wpi (*p* < 0.05, *p* < 0.0001; **Figure 6**). 

### Antibody Titers

**Figure 7:**
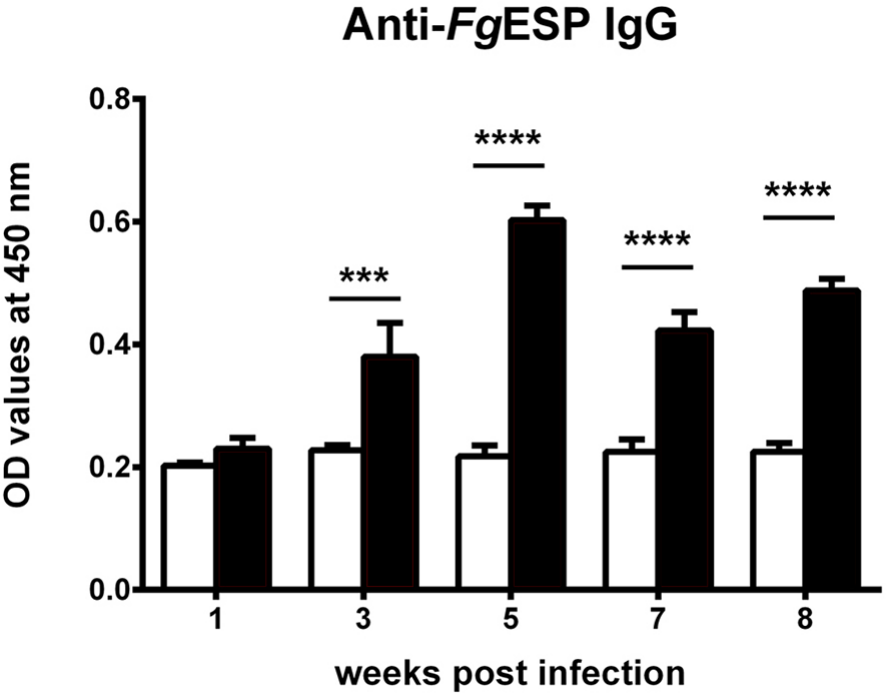
Dynamic changes of serum antibodies in Kunming (KM) mice infected with *Fasciola gigantica* measured using an excretory/secretory product of *F. gigantica* (FgESP)-based direct ELISA method. The *X*-axis represents weeks post-infection (wpi) and the *Y*-axis represents the optical density (OD) values of the antibody titers relative to the uninfected control. *Columns* show the means and *error bars* show SEMs. Significant differences of each time point compared with control: ***p < 0.001; ****p < 0.0001 (analyzed by one-way ANOVA, *post-hoc* LSD test).

Serum specific IgG antibody levels were significantly increased at 3 wpi (*p* < 0.001) and peaked at 5 wpi (*p* < 0.0001). The levels subsequently decreased until the last time point in the experiment (**Figure 7**).

### Correlation Analysis

**Figure 8:**
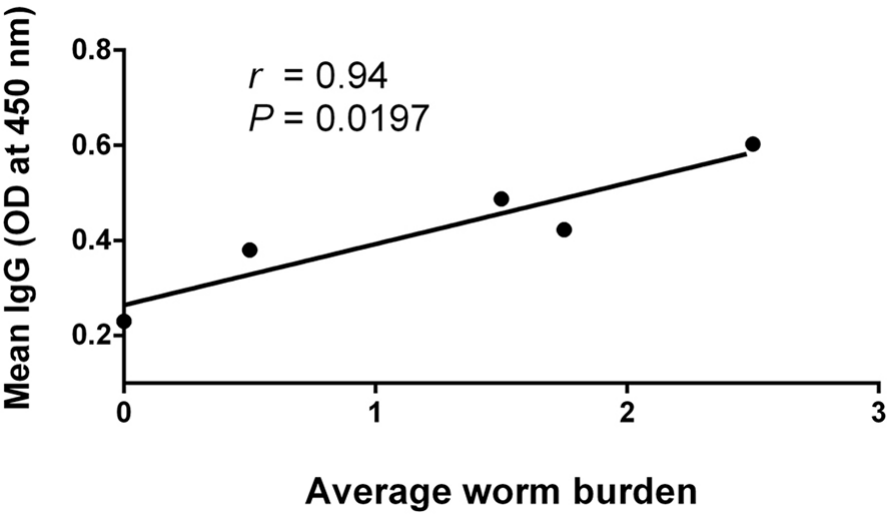
Pearson’s correlation analysis between average worm burdens and *Fasciola gigantica*-specific IgG. Positive correlation between the average worm burdens of infected mice and *F. gigantica*-specific IgG mean levels [optical density (OD) value at 450 nm absorption] at 1–8 wpi of infection based on a Pearson’s linear regression analysis by pairwise comparison. The *solid line* comes from the best-fit linear regression model with a slope of 0.1284, a *Y*-intercept of 0.2640, and a *X*-intercept of −2.055 (*r* = 0.569, *p* < 0.05).

**Figure 9:**
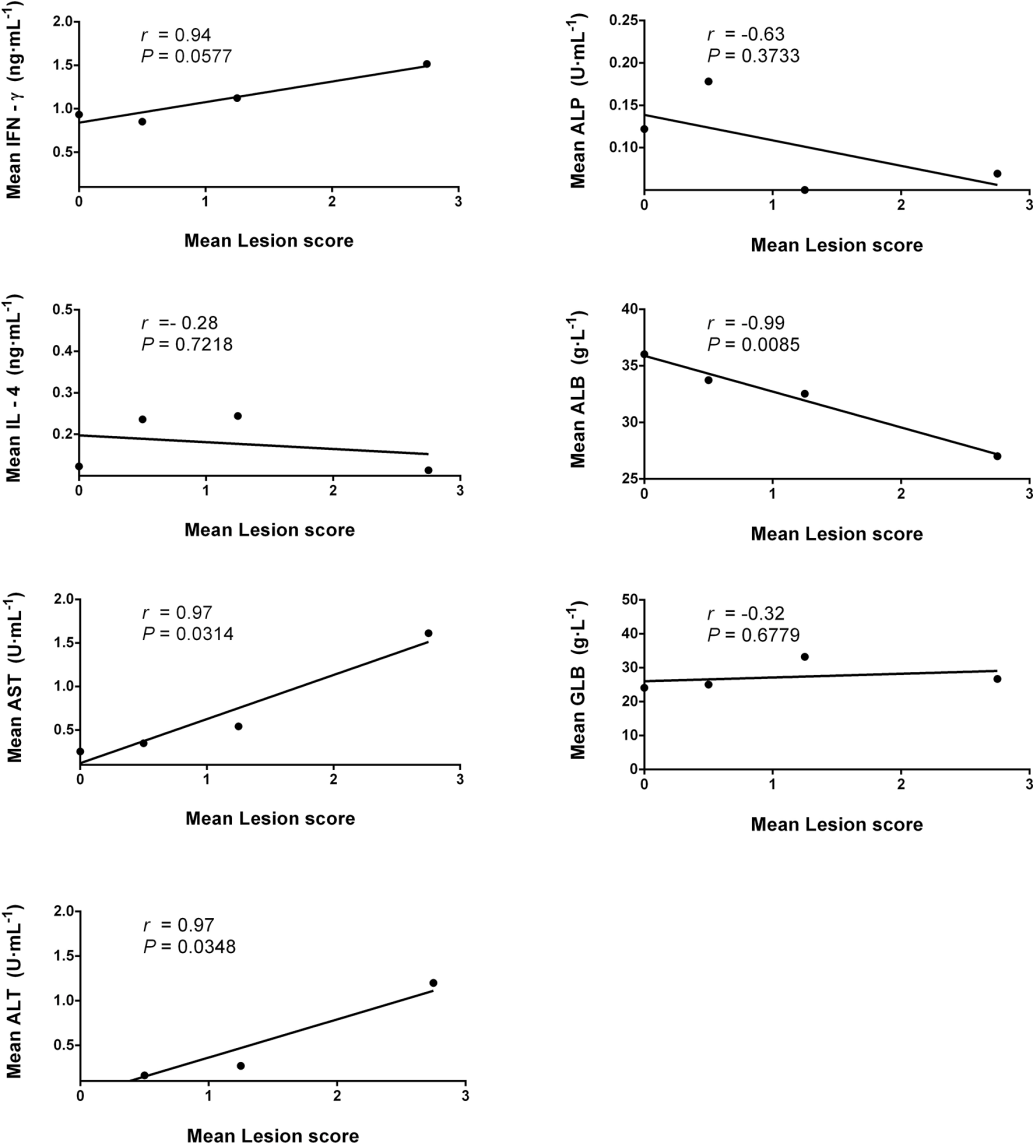
Pearson’s correlation analysis for the mean liver lesion scores of infected mice against the mean values of the serum biochemical indices (AST, ALT, ALP, ALB, and GLB) and serum cytokines (IFN-γ and IL-4) for 1–7 weeks post-infection (wpi) by pairwise comparison. The *solid lines* are from the best-fit linear regression model and illustrate positive (*r* > 0) or negative (*r* < 0) correlation, while *p* < 0.05 represents statistically significant differences. *AST*, aspartate aminotransferase; *ALT*, alanine aminotransferase; *ALP*, alkaline phosphatase; *ALB*, albumin; *GLB*, globulin.

The correlation analysis showed that the serum IgG antibody level was positively correlated with the average parasite burdens between 1 and 8 wpi in the infected mice (**Figure 8**). A possible linear correlation was observed between the macroscopic liver examination scores and the serum levels of IFN-γ, AST, ALT, ALB, and GLB; however, these were not statistically significant (*p* > 0.05; figure not shown). Considering the excessive inflammation and lesion, the data acquired from 8 wpi may not consistently reflect the tendency of each tested indicator. After excluding the 8-wpi data from all tests, significant linear correlations were found between the serum levels of AST, ALT, and ALB and liver lesions between 1 and 7 wpi (*p* < 0.05; **Figure 9**). 

## Discussion

This is the first study evaluating KM mice as a small laboratory infection model for examination of early infection (1–8 wpi) with *F. gigantica*. In this study, we investigated the macroscopic and microscopic changes in infected KM mice, including pathological changes and histopathology in the liver. We also determined and analyzed the dynamic level changes of biochemical indices, parasite-specific IgG antibody, and major Th1/Th2 cytokines in mouse serum. As an essential prerequisite, *F. gigantica* infection was successfully established in KM mice, mainly confirmed by the recovery of parasites at the end. No worm was recovered from infected individuals at 1 wpi. This may be because the size of juveniles at this stage was too small to be seen by the naked eye. Although invisible, this does not entirely mean that the parasites were not present in the tissues.

Serum levels of AST, ALT, ALP, ALB, and GLB can be used as indicators of liver damage (Kolodziejczyk et al., 2005; Kamel et al., 2015; Gattani et al., 2018). AST and ALT are used to evaluate hepatic parenchymal cell injury and are more sensitive than other serum enzymes (Mahmoud et al., 2002; Myers et al., 2003; Pierr et al., 2012; Beek et al., 2013). Elevated ALP levels are common in hepatobiliary diseases and provide important hints about primary biliary cirrhosis (Kaplan and Righetti, 1970; Thapa and Walia, 2007; Lindor et al., 2009). The liver is the largest and one of the most important organs of the immune system. Once the immune cells in the liver are activated, the amount of GLB secreted in the liver increases. Low ALB and high immunoglobulin are commonly seen in fasciolosis (Ulger et al., 2014; Ibrahim, 2017). Previous data suggested that the levels of AST and ALT increased after infection with *Fasciola* in cattle (Edith et al., 2010; Gattani et al., 2018), sheep (Ahmed et al., 2006; Solanki et al., 2017), rats (Kolodziejczyk et al., 2005), and humans (Arslan et al., 2012; Kamel et al., 2015), which is consistent with the results of the current study. Gattani et al. (2018) found that the levels of ALP were elevated in the serum of cattle naturally infected with *F. hepatica.* Moreover, Galtier et al. (2011) showed that rats infected with *F. hepatica* had elevated ALP during the entire chronic phase (when the parasite enters the bile duct). However, the results of our study showed that the levels of ALP significantly decreased at 5 wpi, coinciding with a period of high death rate, which may be related to the anemia and malnutrition observed in mice during this period. Anemia, low ALB, eosinophilia, and subacute and chronic infections characterized by severe weight loss, low ALB, and high GLB are the main clinical features of human fasciolosis (Ibrahim, 2017). A survey in Turkey (Veli et al., 2014) showed that the low ALB presented as one of the symptoms in 44% of *F. hepatica*-infected cases. The results of our study showed that the ALB level exhibited a downward trend and was lower than that of the control group, indicating that, once KM mice were infected with *F. gigantica*, ALB synthesis was reduced due to liver damage. The level of GLB was consistent with the trend of specific IgG antibody levels, meaning that the change in the level of GLB was possible due to the immune responses by mechanical damage and repeated stimulation of the host liver by *Fasciola*-associated proteins.

Due to the diversities in body size and pathogenicity, *F. gigantica* and *F. hepatica* generate different types of cellular immunity when infecting different hosts. For example, cellular immune response shifting from Th0 to Th2 is a characteristic immunological process in cattle infected with *F. hepatica* (Oldham and Williams, 1985). Furthermore, buffaloes infected with *F. gigantica* mainly display a Th0-like immune response (which should now be described as a mixed Th1/Th2 immune response due to the co-upregulation of both serum Th1 and Th2 cytokines observed) (Zhang et al., 2006). In this study, the murine immune response was characterized by a mixed Th1/Th2 response, sharing similar immune response characteristics of large animals. In cattle and buffaloes infected with high parasitic loads (1,000 metacercaria), the levels of IFN-γ in the host sera did not significantly change before and after infection, indicating that these two hosts lack an effective mechanism for killing early larvae (Estes et al., 1994; Hansen et al., 1999; Molina, 2005). In our KM mice, IFN-γ increased at 1 wpi and decreased after reaching a peak at 7 wpi. The specific IgG antibody level reached a peak at 5 wpi and then decreased, while the average parasite burdens were highest at 5 wpi. We infer that a persistent Th1-type inflammatory response in KM mice infected with *F. gigantica* has a deadly effect on the larvae, resulting in the death of a certain percentage of the parasites and a decrease in the total amount of specific IgG antibodies produced. Together with data from large animals (which have a bigger worm burden) and KM mice (which have a lower worm burden), our data suggested that the relatively low worm burden may mainly be due to the early Th1 response characterized by the rapidly increased serum IFN-γ and IgG levels in KM mice. In addition, as we observed in all tested animal models in previous studies, flukes of *Fasciola* spp. could reach over 1 cm in length within 8 weeks in the host (Valero et al., 1998). Obviously, the small size of the bile duct, gallbladder, and hepatic parenchyma might physically (but not immunologically) limit the fast growth and migration of worms inside the hepatobiliary system in mice, unlike that in large animal hosts. This may also be another possible reason for the early clearance of most newly excysted juveniles (NEJs), as well as for the low worm burden after infection of KM mice with *F. gigantica*.

We also observed that the change of serum IL-4 in mice was similar to that of IFN-γ; still, the increase of IL-4 was observed later than that of IFN-γ and remained high for a shorter period than IFN-γ, which is very similar to the immunological response observed in buffalo infected with *F. gigantica* (Shi et al., 2017; Zhang et al., 2017). IL-4 is an important cytokine benefiting liver flukes that contributes to the inhibition of the host Th1-type pro-inflammatory response and is essential for the parasites to achieve immune evasion during the early stage of infection (Cervi et al., 2001). A transient increase of IL-4 from 3 to 5 wpi may contribute to the rapid development and migration of *F. gigantica* at this stage and may have a role in maintaining immunosuppression and promoting tissue fibrosis and repair. Focal fibrosis forms external physical barriers that block the impairment of liver tissues and the access of immune cells to the parasites, thereby creating a protective environment benefiting parasite development (Mendes et al., 2012; Mendes et al., 2013; Shi et al., 2017; Zhang et al., 2017). The production of IL-4 induced by *F. gigantica* infection could promote tissue repair and further liver fibrosis. However, KM mice may not be able to repair the severe liver damage observed, which may lead to more acute symptoms rather than more moderate chronic symptoms during infection. This is evidenced by the occurrence of the first death of infected individuals at 8 wpi, which showed extremely severe liver tissue pathology. We suggest that the main reason for the death could be excessive inflammation and lesion, which gradually increased from 1 to 7 wpi. Similar studies on other animal models (for example, buffalo and sheep) have shown a direct connection between liver damage and parasite burden, as well as infection period in any animal model (Kendall and Parfitt, 1962; Boray, 1967; Prasitirat et al., 1996). Thus, it is clear that the deficiency of tissue repair during longer infection is responsible for the overall damage of the liver in KM mice.

Different infection dosages of parasites may also influence the type of host immune response. O'Neill et al. (2000) found that infection with five *F. hepatica* metacercaria induced a strong Th2 immune response in BALB/c and 129Sv/Ev mice and a mixed Th1/Th2 immune response in C57BL/6 mice. However, infection with 15 *F. hepatica* metacercaria caused the immune response of C57BL/6 mice, making them more Th2-dominant. This is likely closely related to the degree of host organ damage and tissue repair caused by different parasitic burdens. In our study, KM mice infected with 15 metacercaria showed a mixed Th1/Th2 immune response; however, the dynamic changes in cytokine levels during infection with different parasitic burdens must be further confirmed (Machicado et al., 2016).

Detection of serum antibodies using ELISA is the most popular technique for fasciolosis diagnosis (Castro et al., 2000; Reichel, 2002; Molloy et al., 2005; Ridi et al., 2007). Previous studies have shown that parasite-specific serum antibodies in sheep infected with *F. gigantica* increased at 2–4 wpi (Guobadia and Fagbemi, 1995; Phiri et al., 2006; Ridi et al., 2007), while the antibody titers increased at 4 wpi after *F. hepatica* infection (Santiago and Hillyer, 1988). The parasite recovery rate from sheep infected with *F. hepatica* was significantly higher than that from sheep infected with *F. gigantica* (Zhang et al., 2004; Zhang et al., 2005). The delay in antibody production, which prevents the host immune system from completely killing the smaller and more vulnerable larvae during the early stages of infection, is a major factor in the susceptibility of sheep to *F. hepatica* infection. In this study, the level of specific IgG antibody in the serum of mice infected with 15 of *F. gigantica* metacercaria was not significantly changed at 1 wpi, but increased starting at 3 wpi and then remained high until death. This is similar to what has been observed in other susceptible animals (Guobadia and Fagbemi, 1995; Phiri et al., 2006; Ridi et al., 2007), indicating that the production of specific antibodies can be induced at 2–3 wpi in hosts and that the antibody concentration can be maintained until the adult stage (although the experimental host in our case was unable to achieve chronic infection).

Based on the dynamics of various biochemical indices and cytokines, we found that some of these indicators could be associated with the course of progressive host liver disease. Therefore, we further analyzed the correlation between liver lesions and these indicators. Our analysis showed that the liver lesion scores in KM mice were likely to have a linear correlation with IFN-γ, AST, ALT, ALB, and GLB between 1 and 8 wpi; however, the correlation was not statistically significant (*p* > 0.05; figure not shown). In cases where most biochemical and immunological indices were suddenly downregulated at 8 wpi, we assumed that the destruction of liver structures led to a significant decrease in the number of secretory cells. Therefore, the 8-wpi data were intentionally excluded from the correlation analysis, and we found that AST, ALT, and ALB did have a significant linear correlation with liver lesions within 1–7 wpi (*p* < 0.05; **Figure 7**), which again confirmed that AST, ALT, and ALB are reliable and convincing biochemical markers for evaluating the degree of liver injury and the prognosis of hosts from fasciolosis (Edith et al., 2010; El-Boshy et al., 2015), contrary to other indicators. Nevertheless, the lack of correlation between the liver damage scores and the tested serum cytokines suggested that the inflammation balance may not be the major or only influencing factor deciding the degree of liver damage. Herein, our correlation analysis denoted that the continuously intensifying liver damage, but without an effective IL-4-dependent repair mechanism (Minutti et al., 2017; Gieseck III et al., 2018), could be responsible for the liver disease progression of fascioliasis until the final death of the infected mice at 8 wpi. 

## Conclusions

This preliminarily study explored the pathology, biochemistry, and immune responses after early infection of *F. gigantica* in KM mice, characterized as severe and rapid hepatitis. The results of this study will provide a basis for studying the small laboratory animal model of *F. gigantica.* Moreover, measurement of the levels of AST, ALT, and ALB combined with the parasite-specific antibody titer in the serum samples using ELISA can be applied in clinical diagnosis to effectively determine the pathogenic course of *F. gigantica* infection. Understanding of the immunological and biochemical information on KM mice during *F. gigantica* early infection provides an important theoretical framework for further comparative studies on other laboratory animal species (including other mouse genotypes) and may help elucidate the etiology underlying human and animal fascioliasis.

## Data Availability Statement

The original contributions presented in the study are included in the article/Supplementary Material. Further inquiries can be directed to the corresponding author. 

## Ethics Statement

All the animals were housed in an environment with a temperature of 22 ± 1°C, relative humidity of 50 ± 1%, and a light/dark cycle of 12/12 hr. All experimental protocols and methods were approved by the Ethics Committee of the College of Animal Science and Technology, Guangxi University. Animals used in this study were handled in accordance with good animal practices, as required by the Animal Ethics Procedures and Guidelines of the People’s Republic of China.

## Author Contributions

XM, WS, YZ, and WH designed the study and critically revised the paper. XM, WS, YZ, CQ, and YL prepared the experimental samples and performed the experimental procedures. XM, YZ, and WS analyzed the results. XM, WS, and CQ contributed to the writing of the manuscript. All authors contributed to the article and approved the final version. 

## Funding

This study was financially supported by the Doctoral Scientific Research Activation Foundation of Xinxiang Medical University (grant no. XYBSKYZZ202140) and the National Natural Science Foundation of China (grant no. 31760728).

## Conflict of Interest

The authors declare that the research was conducted in the absence of any commercial or financial relationships that could be construed as a potential conflict of interest.

## Publisher’s Note

All claims expressed in this article are solely those of the authors and do not necessarily represent those of their affiliated organizations, or those of the publisher, the editors and the reviewers. Any product that may be evaluated in this article, or claim that may be made by its manufacturer, is not guaranteed or endorsed by the publisher.
